# Emerging Issues and Potential Opportunities in the Rice–Wheat Cropping System of North-Western India

**DOI:** 10.3389/fpls.2022.832683

**Published:** 2022-02-22

**Authors:** Sachin Dhanda, Ashok Yadav, Dharam Bir Yadav, Bhagirath Singh Chauhan

**Affiliations:** ^1^Department of Agronomy, Kansas State University, Manhattan, KS, United States; ^2^Department of Agronomy, Chaudhary Charan Singh Haryana Agricultural University (CCSHAU), Hisar, India; ^3^Department of Agronomy, CCSHAU Regional Research Station, Karnal, India; ^4^Centre for Crop Science, Queensland Alliance for Agriculture and Food Innovation (QAAFI) and School of Agriculture and Food Sciences (SAFS), The University of Queensland, Gatton, QLD, Australia; ^5^Department of Agronomy, CCSHAU, Hisar, India

**Keywords:** environmental pollution, precision management, residue burning, soil health, water productivity

## Abstract

The rice–wheat cropping system (RWCS) is the backbone of Indian farming, especially in the north-western region. But continuous adoption of the RWCS in northwest India has resulted in major challenges and stagnation in the productivity of this system. Additionally, the Indo-Gangetic Plains of Pakistan, Nepal, and Bangladesh are also facing similar challenges for sustainable production of the RWCS. Several emerging problems, such as the exhausting nutrient pool in soil, deteriorating soil health, groundwater depletion, escalating production cost, labor scarcity, environmental pollution due to crop residue burning and enhanced greenhouse gas emissions, climatic vulnerabilities, and herbicide resistance in weed species, are a few major threats to its sustainability. To address these challenges, a wide range of sustainable intensification technologies have been developed to reduce the irrigation and labor requirements, tillage intensity, and straw burning. Awareness and capacity building of the stakeholders and policy matching/advocacy need to be prioritized to adopt time- and need-based strategies at the ground level to combat these challenges. This review summarizes the current status and challenges of the RWCS in the northwest region of the country and also focuses on the precision management options for achieving high productivity, profitability, and sustainability.

## Introduction

The rice (*Oryza sativa* L.)–wheat (*Triticum aestivum* L.) cropping system (RWCS) plays a vital role in global food security as it provides staple foods to the world’s population (Lalik et al., 2014; Banjara et al., 2021a). The RWCS is extensively cultivated and the most technologically advances system in the world. In Asia, 13.5 million hectares (mha) are grown, with 57% of it being in South Asia (Ahmad and Iram, 2006; Ladha et al., 2009). Furthermore, more than 85% of the RWCS practiced in South Asia is distributed in the Indo-Gangetic Plains (IGP; Banjara et al., 2021b). In India, the pre-dominant rice–wheat system covers an area of 9.2 mha, thus playing a key role in the nation’s food security (Jat et al., 2020). India has a remarkable track record of cereal production from 87.4 million tons (mt) in 1961 to 324.3 mt in 2019 (FAOSTAT, 2021). In India, rice occupies an area of nearly 43.8 mha, with a total production of 177.6 mt and productivity of 4,057 kg ha^−1^, whereas, wheat has 29.3 mha area, 103.6 mt production, and 3,533 kg ha^−1^ productivity (FAOSTAT, 2021). Rice is the staple in the diet of >70% of the Indian population, and the rest of the population consumes rice along with wheat or other grains (USDA, 2019).

The RWCS is widely spread in the north-western parts of India, especially Punjab, Haryana, and Uttar Pradesh, and most of these regions depend on groundwater for irrigation (Ambast et al., 2006). With the advent of the Green Revolution, the country’s foodgrain production increased many-fold due to technological intervention; however, current cultivation practices in the RWCS are degrading the soil and water resources and thus threatening the sustainability of this system (Chauhan et al., 2012; Kumar et al., 2018). The crop productivity doubled in the last decade, which happened at the expense of mismanagement of inputs, resulting in a negative impact on the environment, biodiversity, soil quality, and air quality (Tilman et al., 2011; Godfray and Garnett, 2014).

Agriculture is responsible for around 16% of India’s greenhouse gas emissions. Of this, 74% of agricultural greenhouse gas emissions are through methane from livestock (38.9%) and rice (36.9%; Vetter et al., 2017). The remaining 26% comes from nitrous oxide emitted from fertilizers. The RWCS in north-western India is rapidly becoming unsustainable mainly due to rising production cost, depletion/degradation of natural resources, and declining input use efficiencies coupled with changing climate and other socio-economic conditions. There is clear evidence from the long-term experiments that the productivity of the RWCS is stagnating, and sometimes declining (Chauhan et al., 2012; Bhatt et al., 2021). The stagnation in the system yield might be due to an exhausted natural resource base along with adverse effects of changing climate (Pathak et al., 2003). Further, due to the high demand for labor, water, and energy, the sustainability of this system is now under question (Jat et al., 2009; Saharawat et al., 2010; Kumar et al., 2013b; Bhatt et al., 2021).

Historically, rice and wheat have had contrasting requirements for cultivation, as rice seedlings needed to be transplanted under puddled conditions, whereas wheat requires aerobic and well-pulverized soil. However, it is now well-established that rice can alternatively be grown very successfully under non-puddled transplantation or direct-seeding conditions, without a requirement for continuous flooding; similarly, wheat can also be established under no-till conditions (Jat et al., 2019b; Panneerselvam et al., 2020).

In the RWCS, the farmers in north-western India generally grow rice as a lowland crop from June to October, followed by wheat as an upland crop from November to April. The puddling in rice cultivation destroys soil structure, leading to poor soil aeration and soil compaction (Kumar et al., 2008; Pathak et al., 2011). Therefore, the continuous adoption of the RWCS has resulted in a hardpan at shallow depths that halts the root penetration/proliferation into the soil and thus affects the growth of the succeeding wheat crop. A decline in wheat yield (8% lower) was reported when it was sown after puddled transplanted rice compared to the wheat sown after direct-seeded rice (Kumar et al., 2008). The over-exploitation and poor management of groundwater lead to a drastic decline in the groundwater table (Humphreys and Gaydon, 2015).

Rice and wheat are the exhaustive cereal crops that lead to a heavy depletion of soil nutrients, and the problem is further aggravated when farmers burn the rice crop residue left in their fields after mechanized harvesting. The left-over rice residue in the fields interferes with tillage and sowing operations of the successive wheat crop; therefore, farmers usually prefer to burn rice residue. About 2 M farmers in the northwest and some parts of eastern India burn an estimated 23 mt of rice residue every year (NAAS, 2017). Air pollution from crop residue burning is a major cause of premature (human) mortality. In some northwest Indian cities, the particulate air pollution in 2017 exceeded more than five times the safe daily threshold limits causing severe health problems both in rural and urban areas (Central Pollution Control Board of India, 2017). Thus, the residues from the RWCS, especially the rice straw, are a challenge to manage timely and cost-effectively.

**Figure 1:**
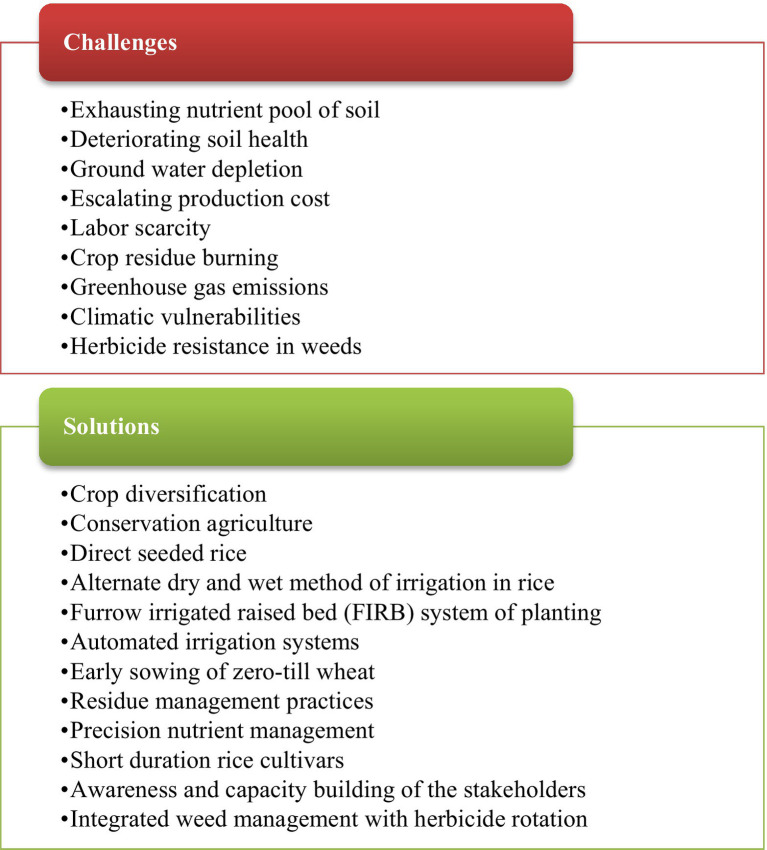
Challenges of the rice–wheat cropping system in northwest India with proposed solutions.

Because of all the challenges posed by the current situation, farmers need new alternatives to the present conventional intensive tillage and crop establishment methods to conserve water and sustain soil health and environmental safety. Also, there is a need to develop new technologies to conserve natural resources and improve input use efficiencies. The challenges and issues with the proposed solutions in the RWCS in northwest India are presented in Figure 1. This review mainly focuses on the importance of the RWCS in northwest India and prevailing and emerging concerns related to continuous adoption of this cropping system *vis-a-vis* alternate sustainable intensification technologies along with precision management options for achieving higher productivity, farm income, and sustainability.

## Significance of the RWCS

The RWCS is the largest agricultural production cropping system in the world and is more prominent in northwest India because of favorable agro-climatic conditions, ecological suitability, and availability of natural resources. Further, the irrigation facilities at nominal charges, assured procurement at a minimum support price, the availability of short-statured and high-yielding fertilizer-responsive as well as irrigation-responsive varieties, and good accessibility to machinery for sowing and harvesting operations favor the adoption of RWCS in northwest India. For these reasons, farmers are reluctant to diversify the existing RWCS. Also, farmers hesitate to shift toward resource conservation and sustainable intensification technologies, like direct-seeded rice, due to the risk of a lower yield than puddled transplanted rice (Kumar and Ladha, 2011).

Moreover, this system plays a major role in sustaining national food security and employs millions of rural people. Also, the RWCS gives stable income with less risk and acts as a mainstay for the livelihood of the farmers and many other stakeholders. Therefore, the regions in northwest India are largely dependent on the monotonous RWCS. In the last few decades, high growth rates for food grain production (wheat 3.0% and rice 2.3%) in India have kept pace with population growth. Rice productivity increased from ~1,000 kg ha^−1^ in 1960 to 3,420 kg ha^−1^ in 2018–19 in Haryana and 4,370 kg ha^−1^ in Punjab. Similarly, wheat productivity increased from 1,200 kg ha^−1^ in 1960–61 to 4,850 kg ha^−1^ in 2018–19 in Haryana and 5,080 kg ha^−1^ in Punjab. However, the productivity growth in rice and wheat slowed down after 2000-01, followed by a widespread stagnation or decline in some locations (Statistical Abstract of Haryana, 2018-19; Statistical Abstract of Punjab, 2019; Haryana Economic Survey, 2019-20; Punjab Economic Survey, 2019-20). Evidence is now appearing that the rice–wheat system productivity is plateauing because of a fatigued natural resource base.

## Traditional Production Practices and Challenges

Conventional rice cultivation in the RWCS is characterized by intensive puddling followed by transplanting of 25- to 30-day-old seedlings, maintaining standing water in paddy fields for 2 weeks, and thereafter re-applying irrigation 2 days after the ponded water has disappeared due to infiltration into the soil (Anonymous, 2020; Dhillon et al., 2021). This practice offers some benefits, such as competitive advantages against weeds and poor oxygen diffusion that restricts the germination of weeds, assured anaerobic conditions with a neutral soil reaction, and greater availability of nutrients, such as iron (Bhatt and Kukal, 2015). The cultivation of puddled transplanted rice is followed with multiple tillage operations to create a fine seedbed for the wheat crop.

The short turnaround time between rice harvest and wheat planting, and farmers’ mindset to employ excessive preparatory tillage primarily delay wheat planting, resulting in yield losses of 32 kg ha^−1^ day^−1^ when planted after 15 November in northwest India (Tripathi et al., 2005), and even as high as 35 kg ha^−1^ day^−1^ in other regions of the country. Wheat sowing can also delay because of the planting of medium duration (140 days) basmati rice or long-duration coarse rice varieties. The fields are kept as fallows for 2–3 months between wheat harvest and rice establishment. This conventional system for the cultivation of the RWCS has led to many challenges, which are discussed below.

### Soil Health

The conventional system for rice cultivation includes puddling (tillage under wet conditions) for reducing the percolation losses, for ease of seedling transplantation, and to suppress weeds. But the continuous adoption of this system has resulted in several negative effects on soil health (Nandan et al., 2021). Repeated puddling in coarse and medium-textured soils has resulted in subsurface compaction, which affects the cultivation of successive upland crops, such as wheat, by restricting the root growth and causing aeration problems (Hossain et al., 2021; Saurabh et al., 2021). Puddled transplanted rice, when following a wheat crop, exposes the hidden organic matter to air and results in its oxidation, thus leading to structural degradation of the soil.

Further, intensive tillage results in the destruction of large aggregates, which ultimately leads to low crop yields. The continuous RWCS is also found to disturb the nutrient balance in the upper vadose zone (Gill, 1992). The burning of rice residues results in loss of nutrients in bulk (Kumar et al., 2019), as a result, farmers have to apply additional fertilizers, which results in a higher cost of cultivation and also reduces the soil quality in the long run. Thus, the continuous RWCS severely affects soil health. A decrease in soil organic carbon of 0.9 t ha^−1^ was reported under a continuous 7-year RWCS in the IGP of India (Sapkota et al., 2017). However, there are divergent opinions regarding the organic carbon and soil fertility under the RWCS.

Bijay-Singh et al. (2005) concluded that the long-term soil fertility experiments on the RWCS progressing in the IGP do not support the sweeping statements that soil organic matter in the entire rice–wheat belt of India is declining. Soil organic matter levels in some areas of the rice–wheat belt are declining only where unbalanced use of fertilizers is being practiced and very little recycling of crop residue and other organic inputs occurs. Yadav et al. (2000) analyzed the data of long-term experiments in the RWCS conducted for 12–15 years at seven locations, which revealed that soil organic carbon decreased overtime at locations where the initial organic carbon content was greater than 0.65%, but increased at locations having low initial organic carbon (<0.50%). Lal (2004) considered lowland rice-based production systems the most stable and soil organic carbon conserving system. However, the RWCS is showing signs of fatigue because of the continuous use of traditional practices which resulted in yield stagnation and declining factor productivity (Bhatt et al., 2016, 2021). Higher yields are being sustained with an increased quantity of nutrients, which is also an indirect indication of deteriorating soil health.

### Residue Burning

Haryana and Punjab together produce 28.1 mt of paddy straw (2018 estimates) out of which 40% (11.3 mt) is burnt in the fields, and 60% is managed through soil incorporation and other measures (DACFW, 2019). These two states of India accounted for 12 and 88% of the straw burnt, respectively (DACFW, 2019). Management of residue is a major problem in the RWCS. The wheat residue is not a major concern as it is used in the animal husbandry sector but rice residue (6–8 t ha^−1^) is not suitable for the dairy sector because of its low digestibility, poor palatability, low protein, and high silica content (Arora and Sehgal, 1999). Also, the incorporation of undecomposed fresh rice straw immobilizes nitrogen in the soil as it has wide carbon: nitrogen ratio (Singh and Sidhu, 2014). Therefore, farmers usually resort to the burning of rice residue, which leads to several problems.

Particulate air pollution from crop residue burning affects the local population and downwind communities. Seasonal rice residue burning is estimated to contribute as much as 26% of the country’s capital’s (Delhi) air pollution in the winter months (Sharma and Dikshit, 2016). The effect of residue burning is evident when an international cricket match was interrupted in Delhi because of high pollution (15 times the World Health Organization limits).^1^ The cricket sport plays an important role in the Indian economy; therefore, residue burning indirectly affects the Indian economy. Even some countries have suspended their flights to Delhi during this pollution phase.^2^

Residue burning imposes enormous public health and economic burden caused by air pollution (Balwinder-Singh et al., 2019). Catastrophic spikes in air pollution are increasingly common in northwest India in the fall season following rice harvest. Such burnings result in perturbations to the regional atmospheric chemistry due to emissions of trace species like carbon dioxide, carbon monoxide, methane, nitrous oxide, and aerosols (Gupta et al., 2004). Fine particulate matter (particulate matter of 2.5) is particularly of concern when values exceed 700 μg m^−3^ in some cities and concentrated burning is the major contributor to poor air quality in northwest India (Balwinder-Singh et al., 2019). Moreover, residue burning causes removal of nutrients, especially potassium, and 80–85% of which is absorbed by rice and wheat residues in their straw (Singh et al., 2008). Therefore, to address all these major issues, scientists have suggested different *in situ* and *ex situ* measures to the farmers and, depending upon their socio-economic and cultural status, one could choose a better option for the judicious use of the straw residue. As an example, the rice straw can be managed by using it as manure or to produce energy, ethanol, biogas, and biochar (Lohan et al., 2018).

### Herbicide Resistance

The continuous use of a single herbicide in rice for the control of *Echinochloa* spp. resulted in the emergence of new hardy weeds like *Leptochloa chinensis* (L.) Nees, *Cynodon dactylon* (L.) Pers., *Ischaemum rugosum* Salisb., *Paspalum distichum* L., *Ludwigia hyssopifolia* (G. Don) Exell, *Eclipta prostrata* L., *Cyperus rotundus* L., *Cyperus iria* L., *Cyperus difformis* L., and *Fimbristylis miliacea* (L.) Vahl (Mahajan et al., 2012; Shekhawat et al., 2020; Dhillon et al., 2021). Due to the monocropping of the RWCS, the infestation of *Phalaris minor* Retz. has increased in wheat, as paddy cultivation provides favorable conditions for the germination of *P. minor* (Franke et al., 2003). Farmers have applied herbicides with similar modes of action year after year, which resulted in strong selection pressure for the resistant biotypes of *P. minor*, and ultimately the spread of resistance increased manifold in many regions of northwest India, especially Haryana and Punjab.

At present, *P. minor* has evolved with multiple resistance to three modes of herbicidal action, namely, acetyl-coenzyme A carboxylase (ACCase), acetolactate synthase (ALS), and photosystem II (PS II) inhibitors (Chhokar and Sharma, 2008; Yadav et al., 2016). Resistance in *P. minor* has been reported to isoproturon, clodinafop-propargyl, fenoxaprop-p-ethyl, sulfosulfuron, mesosulfuron-methyl, iodosulfuron-methyl-sodium, pyroxsulam, and pinoxaden. Farmers at some locations in the Karnal and Kaithal districts of Haryana even applied 3–4 times the recommended dose of herbicides during 2017–18 and ended up with unsatisfactory control (~70%; Singh et al., 2021). Recently, farmers have started using pendimethalin (750–1,000 g a.i. ha^−1^) as pre-emergence and metribuzin (50–140 g a.i. ha^−1^) as post-emergence tank-mix, in addition to the recommended herbicides to get desirable control (Singh et al., 2021). This study also revealed an increase in the cost of cultivation by 6.6% to manage *P. minor* in wheat, and an extra amount of around USD 38 million was spent annually by farmers in the RWCS of Haryana.

In the present situation, the pre-emergence application of pendimethalin, followed by post-emergence application of ACCase or ALS inhibitor herbicides, and tank or premix with metribuzin can control isoproturon and fenoxaprop-resistant *P. minor* (Chhokar and Sharma, 2008; Yadav et al., 2016). The application of pyroxasulfone has been found to be effective in controlling multiple resistant biotypes of *P. minor* (Kaur et al., 2019). As farmers moved toward zero tillage sowing, weed flora also got shifted, and a greater infestation of broadleaf weeds than grass weeds was reported (Chhokar et al., 2007, 2012). The same practice of continuous application of a herbicide with a similar mode of action also resulted in resistance in *Rumex dentatus* L. to metsulfuron-methyl, and cross-resistance to florasulam, pyroxsulam, iodosulfuron, and triasulfuron (Chhokar et al., 2017; Dhanda et al., 2020; Chaudhary et al., 2021).

### Groundwater Depletion

The continuous adoption of the RWCS resulted in a decline in the water table, which ultimately affected crop, land, and water productivity. The Haryana and Punjab states in northwest parts of India are producing rice at the cost of their natural resources (Dhillon et al., 2010), which have been dwindling very fast. The conventional system of rice cultivation is a high-water demanding system (Bhatt and Kukal, 2018; Bhatt et al., 2020). Further, there is a large amount of loss of irrigation water during conventional rice cultivation in the northwest IGPs mainly in coarse-textured soils (Hira, 2009). Since the 1970s, groundwater has been continuously pumped, resulting in its declining level in northwest India, as an aftermath of the Green Revolution (Hira et al., 2004). The mean groundwater depletion in the Punjab state was about 8.91 m between 2000 and 2019 (Sidhu et al., 2021).

The decline in the groundwater table also resulted large investments into tube wells, which increased operational costs and led to more power consumption in northwest India (GoI, 2017), and the deterioration of groundwater quality (Farmaha et al., 2021). The management of groundwater is one of the major priorities to combat the challenging issue of water scarcity in India. Due to an over-reliance on groundwater and poor alternative infrastructure in the northwest IGP, the groundwater levels are declining at 0.1 to 1.0 meters every year (Hira et al., 2004; Bhatt et al., 2020). Based on the Central Ground Water Board report, out of 138 blocks monitored in Punjab, 80% were categorized as overexploited, and the remaining 16% were considered safe (CGWB, 2019). Similarly, in Haryana, 61% of the blocks were categorized as overexploited, and 20% safe. This is primarily because of the rice area expansion over time and continuing high groundwater withdrawal for irrigating rice.

About 92–96% of groundwater withdrawal in Punjab and Haryana is used for irrigation purposes. Because of the declining water table, the farmers are further compelled to deepen their tube wells and switch from centrifugal to submersible pumps, which has further escalated the production cost and challenging the overall sustainability of the existing cropping system. Annual *per capita* water availability in northwest India is supposed to decrease from 1,600 m^3^ to 1,000 m^3^ by 2025 (Mahajan et al., 2011). It was estimated that a reduction in the evapotranspiration from the RWCS of about 150 mm per year was needed to stop this groundwater decline (Humphreys et al., 2010).

### Economics

Rice is a labor-intensive crop, and both Punjab and Haryana are largely dependent on migrant labor for its cultivation. From 2005–06 to 2018–19, both states’ labor costs had increased exponentially from USD 1.27 to 3.78 per day in Haryana, and USD 1.35 to 3.22 per day in Punjab, respectively (1 USD = 74.32 Indian rupees; Sudhir-Yadav et al., 2017). This has increased transplanting costs approximately from USD 47 per ha in 2010 to USD 74 per ha in 2015, and it is rising every year thereafter. In addition, the gap between the overall costs of cultivation and the minimum support prices (proxies of market prices) of rice and wheat crops, even after adjusted for inflation effects, have increased over the years, adversely affecting the overall profitability and farm income. The RWCS, however, still remains the most preferred cropping system in northwest India because of its advantages like assured price and marketing, and stability in yield levels (Bhatt et al., 2021).

Although the RWCS has been sustaining over the years, since the 1990s the productivity showed stagnation (Bhatt et al., 2021), connoting that the pace of productivity from the Green Revolution was slowing down. There is a need for crop rotation, instead of sticking solely to the RWCS. The crop rotation will not only save the resource base, including water, but also give higher economical returns. A study conducted at Ludhiana, India, revealed that inclusion of maize–potato–onion; summer peanut–potato–pearl millet (fodder), and maize–potato–summer mung bean gave 32, 25, and 23 t ha^−1^ annum^−1^ rice equivalent yield, respectively, as against 12.9 t ha^−1^ annum^−1^ in the RWCS (Walia et al., 2011). Substitution of rice with maize or sugarcane was found more profitable than the RWCS (Kang et al., 2009; Choudhary et al., 2018a).

## Alternative Production Technologies and Associated Benefits

For the last three decades, several studies have been concentrating on identifying viable, sustainable, and eco-friendly alternatives to the RWCS (Singh et al., 2020; Banjara et al., 2021b). To improve the deteriorating soil health, reduce groundwater depletion, residue burning, and environmental pollution, and ultimately to enhance farm incomes in a sustainable manner, sustainable agriculture-based technologies need to be adopted at the field level. The inclusion of legume crops in the RWCS, or crop diversification, crop residue management, conservation agriculture, and the adoption of water-saving technologies, can definitely help to increase the land and water productivity in the RWCS in northwest India. Some of the sustainable intensification technologies along with precision management options are discussed below.

### Crop Diversification

There are several benefits associated with crop rotations other than the RWCS. The cultivation of pulses or oilseed crops instead of rice resulted in the improvement of soil health, reduced the water requirement, and increased water productivity (Arora et al., 2020). A study in Punjab, India, revealed that the soybean–wheat system under a raised bed system improved soil fertility and also saved water compared with the RWCS (Ram et al., 2013). Thus, the inclusion of legumes in the cropping system enhanced the nitrogen economy and also contributed to cropping systems’ sustainability (Arora et al., 2020). Conservation agriculture-based opportunistic diversification (e.g., rice–wheat–mung bean) and strategic diversification (e.g., maize–wheat–mung bean) increased soil organic carbon by 83 and 72%, respectively, as compared to rice–wheat (4.6 g kg^−1^; Choudhary et al., 2018b).

Moreover, under a strategic scenario, lower soil bulk density and a higher soil quality index were recorded over the rice–wheat–mung bean system (Choudhary et al., 2018b). The maize–wheat–mung bean and pigeon pea–wheat cropping systems resulted in a significant increase in total soil organic carbon of about 11 and 10%, respectively, as compared to the conventional maize–wheat system (Venkatesh et al., 2017). The substitution of rice with crops that require less water, such as cotton, maize, pearl millet, or legumes in the summer, will allow the water to be replenished during the monsoon season and will increase the water productivity in northwest India. There was a substantial reduction in the irrigation water requirement when the RWCS shifted toward a cotton–wheat or maize–wheat system (Arora et al., 2008).

The maize crop can be a viable alternative to replace puddled transplanted rice in northwest India because of several benefits associated with it. Maize needs 80–85% less water and only one-fourth of water is required to produce 1 kg of grain as compared to rice (Bouman, 2009). The water productivity of maize was 8–22 times higher than rice (Gathala et al., 2013). Similarly, the irrigation water productivity of the maize–wheat system was 126–160% higher than the RWCS. Furthermore, the maize–wheat system also improved the soil health in comparison with the puddled transplanted rice system (Jat et al., 2012). The substitution of rice with maize in the RWCS enhanced the total microbial and enzymatic activities in soil, such as phosphatase, invertase, β**-**glucosidase, and urease (Wei et al., 2015).

It was reported that the activity of alkaline phosphatase and dehydrogenase, and the amount of carbon and nitrogen in the microbial biomass were higher under the maize–wheat–mungbean system than the RWCS (Choudhary et al., 2018b). Governments in north-western Indian states are increasingly interested to diversify the intensified rice–wheat systems, more so to shift from rice to an alternative crop that requires less irrigation water, minimizes residue burning, and reduces labor scarcity. Based on long-term trials in Haryana, India, Kumar et al. (2018) reported that replacing rice with zero-till maize resulted in multiple benefits: 82–89% savings in irrigation water, 49–66% savings in total energy input, 13–40% reductions in global warming potential, and 27–73% higher profitability with similar or higher rice equivalent yield as compared to rice cultivated by transplanting or direct-seeding methods. Similar results were also reported by Jat et al. (2020).

Strategies to manage water demand and reduce crop burning in northwest India include modification in the cropping calendar, a suitable choice of crops and their varieties, and changes in cultivation practices. The prevalent government policies in Punjab and Haryana favor the cultivation of rice and wheat. The government’s attempts to replace rice with less water-requiring crops have not succeeded so far because of policies favoring these two crops, such as subsidized/free electricity for irrigation, assured output markets, minimum support price (MSP) guarantees, and a lack of suitable and remunerative alternative crops for diversification. The government has started to focus on pulse crops with strategically raising the MSP in the recent years. Alternate policies with strong advocacy could encourage farmers to downsize the stretch of rice–wheat farming in these states and diversify to more for high-value crops, such as vegetables, fruits, and flowers suited to specific environments.

### Crop Residue Management

Crop residue management in the RWCS is a major challenge because the loose and scattered residue causes hindrance in tillage operations and wheat sowing through conventional tillage becomes difficult and energy-intensive. The farmers in northwest India opt for the burning of residue eventuating in several hazards. For the last so many years, different innovative rice residue management strategies have been developed including *in situ* residue incorporation and zero-till sowing of wheat with surface-retained rice residue. These technologies have many benefits over rice residue burning, such as improving soil health, creating a positive nutrient balance in the soil, decreasing environmental pollution, and ultimately lowering the cost of cultivation.

Singh et al. (2019) reviewed crop residue management in the RWCS and outlined its importance for resource conservation, environmental protection, and sustainability in northwest India. Rice residue burning was monitored by using multiple satellites with thermal sensors during the rice harvest and wheat sowing season period from 30 September to 30 November in northwest India, mainly in Punjab, Haryana, and Uttar Pradesh, and it was found that in Punjab there was about 11 and 42% reduction in the number of burning events in 2018 as compared to that in 2017 and 2016, respectively (Singh et al., 2019). The reduction in number of burning events was about 29 and 41% in Haryana and 24 and 32% in Uttar Pradesh in the corresponding years. The adoption of *in situ* crop residue management practices was found to be the best remedy against crop residue burning.

The development of the zero tillage drill enables the direct sowing of wheat without preparatory tillage and removal of rice residue from the fields. Further, the improved version of zero tillage drills with the Turbo Happy Seeder can sow the wheat seed directly in the standing and lose rice stubbles, just after the harvesting of rice (Sidhu et al., 2015). These inventions are economically viable and increase water use efficiency. Different types of machinery available with the farmers to manage the rice straw include seeders for crop sowing under rice straw conditions (rotary disc drill, Turbo Happy Seeder, zero-seed drill, spatial no-till drill, and super seeders), straw cutter machinery for *in situ* incorporation/retention of rice straw (rice straw chopper, straw shredder, super straw management system and mulcher), machinery for straw incorporation (reversible moldboard plow and rotary-till-drill), and straw collection and disposal (raker and baler). The use of straw for biochar, biogas, and other forms of energy are slowly gaining importance but the considerable investment and time required to setup these enterprises is a major reason for the slow rate of shift of farmers. A delineation of a complete set of practices is lacking for the succeeding crops, such as wheat, potato, and/or vegetables.

### Sustainable Intensification Technologies

Conservation agriculture has emerged as a promising technology to get maximum efficiency of the inputs and to sustain the productivity of the RWCS. Conservation agriculture works on the three fundamental principles, namely, crop diversification, zero or reduced tillage, and residue retention (Farooq and Siddique, 2014), and can help in carbon sequestration and improvement in physicochemical properties of soils (Jat et al., 2019a,b). Studies revealed that practicing sustainable intensification technologies in conservation agriculture systems have resulted in soil health improvement after 3–5 years under the RWCS in the northwest IGP (Bhatt and Kukal, 2015). Further, the retention of residue also helps conserve soil moisture, regulate soil temperature, and enhance water use efficiency (Kukal et al., 2014; Bhatt and Kukal, 2018).

Dry direct-seeded rice (DDSR) has evolved as a potential alternative to puddled transplanted rice. In the DDSR system, the seeds of rice are sown with or without tillage and irrigation. Irrigation is applied at certain intervals to maintain the soil at the field capacity. Between 11 and 18% of water can be saved in the DDSR system, and the labor requirement is reduced by 11–66% when compared to puddled transplanted rice, depending on the season and location (Kumar et al., 2009; Rashid et al., 2009). Further, DDSR also provides other advantages, such as easier and smoother planting, better soil health, and less methane emission (Pathak et al., 2009; Kumar and Ladha, 2011). Rice grown using the DDSR system also matures 7–10 days earlier compared to puddled transplanted rice, and thus allows timely planting of the successive wheat crop (Singh et al., 2006). But the major challenge in DDSR is the potential for a higher infestation and more diverse flora of weeds than puddled transplanted rice (Rao et al., 2007; Singh et al., 2007; Chauhan, 2012). Weeds in the DDSR system can cause a yield loss of 50–90% (Chauhan and Johnson, 2011; Chauhan and Opena, 2012).

During the initial phase of the DDSR (2010–15), the rate of adoption was very good in northwest India. It was reported that the area under DDSR in Punjab was once reached as high as 160,000 ha during 2016, but then declined to 5,000 ha by the year 2018 (Dhillon and Mangat, 2018). The major reasons for the declining area under DDSR were the lack of suitable rice varieties for late planting, too wet soil for dry seeding, and high weed infestations, which led some farmers to plow the crops. But due to labor shortage (migration) because of the lockdown in India in response to the COVID-19 pandemic, the area under DDSR was estimated to be increased to 200,000–250,000 ha in Punjab during 2020 (Humphreys and Christen, 2020). Actually, it was reported that the area under DDSR in Punjab was 500,000 ha in 2020.^3^ Enthusiastic with such a response by farmers, the Punjab state government has set a target of 0.8 m ha in 2021 (Kaushal, 2021), and the latest estimates indicate the DSR area in Punjab and Haryana during 2021 is around 0.6 and 0.02 mha, respectively.

Similarly, the planting of wheat under zero tillage after the rice harvesting had been widely adopted by the farmers in northwest India, especially Punjab, Haryana, and Uttar Pradesh. The area under zero-till wheat after the partial burning of the rice straw significantly increased since the late 1990s because of the reduction in the cost of cultivation by excluding preparatory tillage along with higher yields associated with timely sowings (Gupta and Sayre, 2007). Additionally, zero-till sowing also improved soil quality, prevented soil erosion, enhanced soil microbial activities, reduced weed infestation, reduced termite attack, and increased resource-use efficiency (Hobbs et al., 2008). The direct sowing of wheat with a zero-till seed drill in the combine-harvested rice fields is a challenge because loose rice straw in narrow swaths interferes with wheat sowing, resulting in non-uniform placement and germination of seeds.

This problem has been largely resolved by the development of new machinery, such as turbo Happy Seeder and rotary disc drill (Sidhu et al., 2007), which help by simultaneously surface mulching the rice straw while directly sowing the wheat under no-till conditions. Zero-till wheat was recommended during 2002–2005 in northwest India and now more than 2,500 and 3,400 numbers of zero-till seed-*cum*-fertilizer drills are functional in Haryana and Punjab, respectively, contributing to wheat seeding largely without any tillage or at least with reduced/conserved tillage (1–2 plowings in place of the 4–6 plowings that were commonly employed under conventional tillage).

The Happy Seeder was commercially introduced in northwest India almost 15 years ago. After multiple modifications, this technology has also been successfully adopted by the farmers. As per the 2018–19 report, approximately 2,400 and 9,800 units of Happy Seeder are functioning in Haryana and Punjab covering an area of 0.053 and 0.45 m ha of wheat, respectively (Singh, 2018). Overall, the area coverage under DDSR and zero-till wheat/Happy Seeder in India has been reported to be around 7.2 m ha (Pradhan et al., 2018) and 0.8 m ha (Singh, 2018), respectively.

Super seeders, like a combined version of the Happy Seeder and a rotavator, are also in place to accomplish wheat sowing in fields with full rice residues in one pass, but have a higher energy demand (50–60 H.P. tractor) and fuel consumption (~20 L ha^−1^). Additionally, non-synchronous and non-uniform germination, poor tillering, higher nutrient demand to ameliorate their deficiencies, non-synchronous crop maturity, and ultimately a significant decline in wheat yields are adverse effects associated with this intervention. Similar incidences in wheat have already happened with rotavators. Consequently, the farmers are also gradually losing their interest in wheat sowing by using super seeders.

The Punjab and Haryana state governments have now made it compulsory for all self-propelled combine harvesters to have a “Super Straw Management System,” which helps in chopping and spreading the straw from the harvester. The uniform spreading of the loose residues is very important for a uniform and good crop stand using the Happy Seeder. It was reported that sowing wheat with the turbo Happy Seeder under rice residue conditions increased the wheat yield by 3.2% in comparison with conventional till sowing (Sidhu et al., 2015; Bhatt et al., 2021). The zero-till sowing of wheat with the retention of rice residue enhanced the productivity of the RWCS along with creating a positive nutrient balance and improved soil health (Sah et al., 2014).

The governments of Haryana, Punjab, Uttar Pradesh, and Delhi are helping the farmers by providing a subsidy (50% to an individual farmer and 80% to co-operative societies) on agricultural machinery for the rice straw management and to control the pollution due to residue burning. Also, the governments are organizing awareness campaigns through demonstrations, information dissemination, school curriculum, and using different tactics of information and communication technology for effective management of crop residues. The increase in the number of service providers, individuals, or through custom hiring centers, assisted by several government subsidy schemes, has enhanced farmers’ access to these machines.

Based on the data available from the Punjab Remote Sensing Centre (2019), the area under Happy Seeder sown wheat crops in Punjab was 0.55 m ha while it was not possible for remote sensing technology to assess the area under residue incorporation. It was estimated that nearly 0.8 m ha (19%) of the area was sown under zero-till wheat (using Happy Seeder and zero-till drill) in Punjab and Haryana together during 2018–19. For getting the maximum benefit of zero tillage, both rice and wheat need to be grown in zero-till conditions (Bhushan et al., 2007). However, DDSR in moist soil (sowing in well-prepared fields after pre-sowing irrigation or rains) has been found to be promising across landscapes and ecosystems, and it is gaining increasing importance in Punjab and Haryana, more particularly due to huge labor shortage (migration) during summer seasons of 2020 and 2021 under the effect of COVID-19 pandemic (Workie et al., 2020; Kaur and Kaur, 2021).

The sustainable production of the RWCS includes zero tillage with the retention of crop residue and the inclusion of legumes and *Sesbania* green manure in the system (Bhatt and Kukal, 2018; Yadav et al., 2019). The practice of conservation agriculture with precision irrigation management and replacing rice with low water demanding crops can help to achieve the goal of sustainable crop production in northwest India. It was reported that zero-till DDSR, followed by zero-till wheat, followed by zero-till mung bean resulted in higher system productivity (15–17%) as compared to conventional puddled transplanted rice followed by conventional wheat (Kumar et al., 2018).

Due to other concerns (nematodes, insect pests, and lower yields) associated with long-term zero-till DDSR, dry seeding in moist soil is now more preferred and a potential alternative across the northwest and eastern ecologies. This helps in saving irrigation water, labor, fuel, cost, and drudgery, besides resulting in higher system productivity (more yield of the succeeding crop in sequence), conservation of natural resources, and environmental safety through reducing the emission of methane (>90%) and overall global warming potential (20%).

The DDSR followed by zero tillage wheat (ZTW) on long-term basis may be a viable and economical alternative to the conventional rice–wheat system. A medium term (7-year) study on DDSR-ZTW in Haryana, India was realized a better alternative in cutting cost of cultivation (USD 448 ha^−1^) as compared to the puddled transplanted rice in sequence with conventional till wheat (USD 555 ha^−1^; Yadav et al., 2021). In rice, there was a saving in preparatory tillage (USD 55 ha^−1^), seed/nursery establishment (USD 55 ha^−1^), irrigation cost (USD 29 ha^−1^), and saving of human labor (25 man-days ha^−1^) under DDSR-ZTW; however, weed management cost got increased due to dependence on costly herbicides (Table 1).

**Table 1:** Variable cost of cultivation and labor use in rice under dry direct-seeded rice-zero-till wheat (DDSR-ZTW) in comparison with puddled transplant rice-conventional till wheat (PTR-CTW) in the rice–wheat cropping system in Haryana India (average of 2010–2016).

Component	Cost (USD ha^−1^)	
	DDSR-ZTW	PTR-CTW
Preparatory tillage	56	81
Seed/Nursery/Establishment	55	113
Irrigation	96	125
Weed management	53	15
Total variable cost of cultivation	448	555
Labor use (No. ha^−1^)	56	81

Source: Yadav et al. (2021).

The emission of methane from the soil in puddled transplanted rice ranged from 0.8 to 1.9 t CO_2_ equivalent ha^−1^ in various districts of Punjab compared to only 0.1–0.3 t CO_2_ equivalent ha^−1^ in DDSR (Gartaula et al., 2020). The average global warming potential due to all the three greenhouse gases (carbon dioxide, methane, and nitrous oxide) in transplanted rice was 2.91 t ha^−1^ compared to 1.94 t ha^−1^ in DDSR (Gartaula et al., 2020). Gupta et al. (2016) reported significantly low methane emission (82–87.2%) in the DDSR as compared to the puddled transplanted rice. DDSR leveraged with short or medium duration rice varieties/hybrids with early maturity and faster field vacation helps in conserving residual soil moisture useful for crops in rotation, widening the time window for effective residue management, and also facilitates in early or timely sowing of long-duration wheat varieties ultimately leading to enhanced system productivity, profitability, and sustainability. However, to achieve accelerated and wider scale adoption, concerted efforts are required to create awareness, improve the skills of farmers, and create strong policy support.

### Water-Saving Technologies

Real water saving is referred to as the minimization of the water losses and applying water which is just necessary for plant growth. The amount of water saved can vary, based on the spatial and temporal scales of interest. The water-saving in the RWCS refers to an increase in productivity using less water than is presently being used. The saving of water has additional benefits also in terms of reducing the cost of cultivation (pumping and water charges) and increasing water productivity. The major drawback with puddled transplanted rice is the consumption of excess water, leading to low water productivity. Therefore, different water-saving technologies can be useful in enhancing water use efficiency. These technologies include the cultivation of short-duration crop varieties and transplanting them at the optimum time (Sharma et al., 2020; Singh et al., 2020).

The tensiometer-based irrigation scheduling, laser land leveling, direct seeding of rice, bed planting, subsurface drip irrigation system, furrow irrigation, and alternate wetting and drying for rice or applying irrigation at hairline cracking are some of the major technologies for efficient water use in the RWCS. The micro-irrigation system aims to reduce water usage by avoiding surface runoffs and thus improving efficiency of irrigation by 50% in the RWCS as compared to flood irrigation (Sidhu et al., 2019). It also provides opportunity for provision of nutrients *via* fertigation. Coupled with automatic irrigation practices, it considerably reduces the number of days of labor requirement and hence lower the production cost. The shift from the puddled transplanting to DDSR saves a large amount of water (20–30%) and eliminates the need for irrigation for puddling and transplanting. Similarly, the alternate wetting and drying system of irrigation largely reduces the irrigation requirement (30–40%) after crop establishment both in puddled transplanting as well as direct seeding in rice. The inclusion of short-duration rice cultivars, instead of long ones, increases the water use efficiency by reducing the evapotranspiration losses (Singh et al., 2020). Sprinkler and drip irrigation in DDSR are also potential and emerging opportunities to save irrigation water (Chauhan and Abugho, 2013; Sharda et al., 2017).

Drip irrigation (surface and subsurface) systems provide water and nutrients to the crop root zone, where it can be utilized most effectively. Automation of irrigation in general and drip irrigation in particular with use of sensor network and communication technologies can help in addressing the emerging challenges of inefficiency of water use in the agriculture (Sidhu et al., 2021). Multiple cropping system of summer mung bean–maize–wheat as alternative to the rice–wheat system, enabled by subsurface drip irrigation and fertigation techniques could lead to 30% savings in irrigation water (Brar et al., 2021). Also, optimizing irrigation may help to reduce methane emissions and net global warming potential in the rice–wheat cropping system (Sapkota et al., 2020).

Furrow irrigation in raised bed could also be an alternative to flood irrigation in the field crops to increase water productivity, particularly in the upland crops. Kumar et al. (2010) reported that by following furrow irrigated bed planting systems in wheat, on an average 40% water was saved as compared to flat planting with significant increase in water productivity. Kumar et al. (2013a) reported that furrow irrigated raised bed in the rice–wheat system increased wheat yield and yield attributing characters, but rice crop yield and yield attributes did not differ significantly in raised bed and conventional establishment method; however, root growth of rice was more in bed transplanting. There is a scope for raising rice also on raised beds; however, reports of saving water in rice are lacking. However, furrow irrigated raised beds do not always save irrigation water or increase water productivity in comparison with conventionally tilled flat fields, for both rice and wheat (Kukal et al., 2010), and seems to have limited potential under the prevailing soil and climatic conditions of Punjab, India (Singh et al., 2009). There is a scope of its refinement for better outcomes and that warrants for continued research across landscapes.

### Soil Health Improvement

The fields which witness loss of soil organic matter due to puddling can be fortified with the use of farmyard manure for which the raw materials readily available. *In situ* crop residue management for rice stubble with above-mentioned machines as well as with microbial decomposers can also help to improve soil organic matter content. Use of bio-fertilizers also presents a unique opportunity to reduce the dependence on fertilizers in the RWCS (Khan, 2018).

Site-specific nutrient management (SSNM) with use of Nutrient Expert^®^ (NE) can be employed for improved productivity and nutrient use efficiency in the RWCS. No-tillage system along with SSNM can increase yield, nutrient use efficiency, and profitability while decreasing greenhouse gases from wheat production in the NW India (Sapkota et al., 2014). The adoption of SSNM significantly increased the net returns by 34 to 43 USD ha^−1^ over farmers’ practice of fertilizer application by saving fertilizer input cost (Parihar et al., 2020). Optical sensor-based SSNM saved 20–30 kg N ha^−1^ with similar yield under conservation agriculture-based cereal systems compared to recommended dose of fertilizers. Efficient management of N fertilizers also reduced nitrous oxide emissions by avoiding N losses *via* volatilization, leaching, and denitrification. SSNM provides opportunities for enhancing crop productivity, profitability, and nutrient use efficiencies across the different ecologies. Drip irrigation system (subsurface drip irrigation) also helps in improving the N-use efficiency by 20% over flood irrigation in the rice/maize-based systems (Jat et al., 2019c).

Integrated nutrient management (INM) could help in maintaining soil health and nutrient balances in the RWCS. Data from long-term rice–wheat cropping sequence (30th cycle) indicated that highest rice and wheat grain yields were obtained when 50% N supplied through green manure and farm yard manure, respectively. The soils did not suffer loss of K and N where organics replaced 50% N (Saha et al., 2018). Inclusion of Sesbania green manure in the RWCS may help in sustainable higher productivity with improvement in soil fertility parameters like bulk density, organic carbon, phosphorus, and potassium (Yadav et al., 2010, 2019). Conservation agriculture-based intensification of the RWCS with inclusion of summer mung bean can also improve the soil chemical and biological properties and sustainability of the system (Jat et al., 2016).

### Suggested Policy Changes

A focus on improving data availability for crop succession following *in situ* residue management can help in promoting the use of machinery. Furthermore, an emphasis on promoting crop diversification with increasing the Minimum Support Prices for other relevant crops may serve as a positive reminder to the farmers. Subsidy and demonstrations for micro-irrigation equipment can be popularized to promote their usage. The government should also focus on providing infrastructure for *ex situ* utilization of farm stubble like bio-ethanol plants, biogas plants etc. To monitor soil health, testing campaigns can be organized in the villages to spread awareness about advantages of soil testing.

## Conclusion

Northwest India contributes a major portion in the production of rice and wheat and fills the increasing empty stomachs. But with time, the RWCS has faced many sustainability issues, such as depletion of soil fertility, declining ground water table, and environmental degradation. Therefore, there is a need to shift from the conventional RWCS to conservation agriculture and adopt the need-based practices for the sustainable production of crops. More emphasis should be given to developing high-yielding and short-duration rice varieties, which can help in reducing evapotranspiration loss and produce less residue load at harvest. The inclusion of short-duration legumes, like mung bean, can greatly help in increasing the cropping system rice equivalent yield. Less water-demanding crops, such as maize, in place of rice also needs to be explored for sustainable agriculture production. Automation of irrigation methods can help in addressing the emerging challenges of inefficiency of water use in agriculture. Scaling of sustainable intensification technologies (DDSR and zero-till wheat) by strategically leveraging with suitable varieties, creating more awareness on improved management practices by strengthening the existing extension system, and attracting policy support are needed to achieve an enhanced farm income and sustainability in northwest India.

## Author Contributions

SD and AY: conceptualization. SD, AY, and DY: writing-original draft. SD, AY, DY, and BC: writing-review and editing. AY, DY, and BC: checked and corrected the final draft. All authors contributed to the article and approved the submitted version.

## Conflict of Interest

The authors declare that the research was conducted in the absence of any commercial or financial relationships that could be construed as a potential conflict of interest.

## Publisher’s Note

All claims expressed in this article are solely those of the authors and do not necessarily represent those of their affiliated organizations, or those of the publisher, the editors and the reviewers. Any product that may be evaluated in this article, or claim that may be made by its manufacturer, is not guaranteed or endorsed by the publisher.

## Footnotes

^1^ www.theguardian.com/world/2017/dec/03/pollution-stops-play-at-delhi-test-match-as-bowlers-struggle-to-breathe

^2^ www.hindustantimes.com/delhi-news/delhi-air-pollution-united-airlines-suspends-flights-to-capital-due-to-poor-air-quality/story-srziMMH6HtVpy6rcNcQpHN.html

^3^ www.pau.edu/index.php?_act=manageEvent&DO=viewEventDetail&intEventID=5271
